# Dynamic Doppler Ultrasound Assessment of Tissue Perfusion Is a Better Tool than a Single Vessel Doppler Examination in Differentiating Malignant and Inflammatory Pancreatic Lesions

**DOI:** 10.3390/diagnostics11122289

**Published:** 2021-12-07

**Authors:** Przemysław Dyrla, Arkadiusz Lubas, Jerzy Gil, Marek Saracyn, Maciej Gonciarz

**Affiliations:** 1 Department of Gastroenterology, Military Institute of Medicine, 04-141 Warsaw, Poland; jgil@wim.mil.pl (J.G.); mgonciarz@wim.mil.pl (M.G.); 2 Department of Internal Medicine, Nephrology and Dialysis, Military Institute of Medicine, 04-141 Warsaw, Poland; alubas@wim.mil.pl; 3 Department of Endocrinology and Isotope Therapy, Military Institute of Medicine, 04-141 Warsaw, Poland; msaracyn@wim.mil.pl

**Keywords:** pancreatic tumor, Doppler ultrasonography, tissue perfusion

## Abstract

Dynamic tissue perfusion measurement (DTPM) and single vessel flow measurement (SVFM) were assessed in differentiating inflammatory and malignant lesions of the pancreas. Sixty-nine patients (age 62.0 ± 14.7; 33 Female and 36 Men; 40 with malignant and 29 with inflammatory lesions) in whom during the endoscopic ultrasound (EUS) of focal pancreatic lesions it was possible to adequately evaluate the flow in the color Doppler, and then perform a biopsy, were qualified for the study. The assessed DTPM parameters flow velocity (TFV), perfusion intensity (TPI), and resistive index (TRI) as well as the following SVFM parameters: flow velocity (FV), volume flow (VolF), and resistive index (RI) differed significantly between the malignant and inflammatory lesions (*p* < 0.005). TFV and TPI have slightly better discriminatory properties than the corresponding FV and VolF parameters (*p* < 0.10). Considering the Doppler parameters usually evaluated in a given method, the TPI = 0.009 cm/s (sensitivity 79%, specificity 92%, AUC 0.899, *p* < 0.001) was significantly better (*p* = 0.014) in differentiating between inflammatory and malignant pancreatic lesions in comparison to FV = 2.526 cm/s (sensitivity 79%, specificity 70%, AUC 0.731, *p* < 0.001). Tissue perfusion has better discriminatory properties in the differentiation of solid pancreatic lesions than the Doppler blood flow examination in the single vessel within the tumor.

## 1. Introduction

Pancreatic cancer (PC) is one of the most malignant tumors [1,2]. Most of the patients at the time of diagnosis have advanced PC, which is unresectable. The 5-year mortality in patients with PC is still high, and the survival is poor [3]. The risk factors for pancreatic cancer include a history of the disease in the family, cigarette smoking, chronic pancreatitis, obesity, and diabetes mellitus. PC occurs most frequently in the 60 to 80 year age group, and its incidence is 50% higher in men than in women and individuals with genetic mutations [4]. It is a disease with an overall 5-year survival rate of less than 8%, as presently early detection methods or effective treatments are unavailable [5].

In previous works, with the use of external software, we showed the possibility of differentiating inflammatory pancreatic lesions from PC using organ perfusion parameters evaluated on the basis of the color Doppler [6,7]. Tissue perfusion flow velocity proved to be the best parameter differentiating these two most common types of pancreatic tumors. In view of the above results, the question arises whether the option of assessing flow velocity only in a single tumor vessel using color and pulse Doppler is also useful and allows for an adequate distinction between inflammatory and malignant lesions already during ultrasound examination. This solution would allow for a faster diagnosis without recording of movie sequences with their external evaluation using additional software.

This study aimed to compare the discriminatory properties of two methods of flow assessment, tissue perfusion and a single vessel examination, in differentiating inflammatory and malignant pancreatic lesions.

## 2. Methods

Seventy-seven patients with solid pancreatic tumors diagnosed in the Gastroenterology Department were qualified for this prospective, single-center study. Each patient was over 18 years of age and agreed to the examination and biopsy. Focal lesions of the pancreas were evaluated using the color Doppler, and then a biopsy was performed under EUS control.

Exclusion criteria were no consent for the study, no consent for pancreatic tumor biopsy, and no vessel visible within the lesion in color Doppler.

Endoscopic ultrasound (EUS) examination (Pentax EG-3870 UTK, 5–12 MHz linear transducer) was executed with 2D, and color Doppler imaging, as well as EUS Fine Needle Aspiration (EUS-FNA) using EchoTip/ProCore needles, was performed to collect samples for cytology/histology.

### 2.1. Doppler Flow Assessment

In EUS, color Doppler was used to visualize the blood flow in the pancreatic tumor. First, 3–5 s movie sequences with color Doppler imaging, identifying the blood flow in at least one vessel within the lesion, were recorded. Sequences containing artifacts and/or calcifications that did not allow for an adequate assessment of the blood flow within the lesion (3 patients with FNA-confirmed pancreatitis), as well as sequences showing a relatively large vessel running through the lesion without significant direction modeling (large correct vessel surrounded by the tumor tissue) (5 patients with diagnosed adenocarcinoma) were excluded from further analysis. Eventually, 69 patients with pancreatic tumor qualified for the study (age 62.0 ± 14.7; 33 Female, 36 Men; 40 patients with the pancreatic adenocarcinoma and 29 with the inflammatory tumor).

Dynamic tissue perfusion measurement (DTPM) was performed by determining the region of interest within the color Doppler scanning gate, covering the largest possible tumor area using a dedicated medical device for organ perfusion assessment (PixelFlux, Chameleon-Software, Leipzig, Germany). Automatic analysis of frames in each movie sequence allowed the average (arterial and venous) tissue flow velocity (TFV [cm/s]), average tissue resistive index (TRI [ratio] = (maximal velocity − minimal velocity)/maximal velocity), and average tissue perfusion intensity (TPI [cm/s] = (average velocity × vascular area)/region of interest area) to be obtained [8]. The transfer of 1 patient’s movie file from the ultrasound machine to the PC and the flow assessment in the PixelFlux (DTPM) medical software together lasted approximately 6–7 min.

Single vessel flow measurement (SVFM) was performed by identifying the largest vessel with a known flow direction within the lesion, setting the region of interest, and selecting the automatic angle correction option in the PixelFlux program. Through the automatic movie sequence analysis, the results of the single vessel flow velocity (FV) [cm/s], resistive index (RI), and volume flow (VolF) [mL/s] were obtained.

### 2.2. Statistics

Statistical evaluation was performed using the Statistica 12 package (StatSoft Inc., Cracow, Poland). The results of the measurements are presented as means with the standard deviation or median with the interquartile range depending on the fulfillment of the condition of normal distribution. In the case of parametric variables, the differences between the means of both groups were examined using the T-Student Test for unrelated variables; otherwise, the Mann–Whitney U Test was used. Discriminatory properties for variables were assessed using ROC curves.

## 3. Results

Patients with malignant and inflammatory pancreatic lesions did not differ in age (63.2 ± 16.5 vs. 60.3 ± 11.9; *p* = 0.418). All pancreatic tumor blood flow parameters evaluated by both methods differed significantly between the groups (Table 1).

In SVFM, the mean correction of the Doppler angle to the vessel axis was 48.7 ± 19.1°, and in each case, the flow spectrum was characterized by a large systolic-diastolic difference suggesting arterial flow. The discriminatory properties of organ perfusion parameters and blood flow in a single vessel of a pancreatic tumor are shown in Table 2.

Conversely, the discriminatory properties of TVF, TPI, FV, and VolF with their cut-off point values were the same for recognizing inflammatory from malignant pancreatic lesions. Only nadir values of TRI (0.891, sensitivity 0.793, specificity 0.850; AUC 0.831; *p* < 0.001) and RI (0.865, sensitivity 0.586, specificity 0.850; AUC 0.698; *p* = 0.003) were slightly changed.

**Figure 1:**
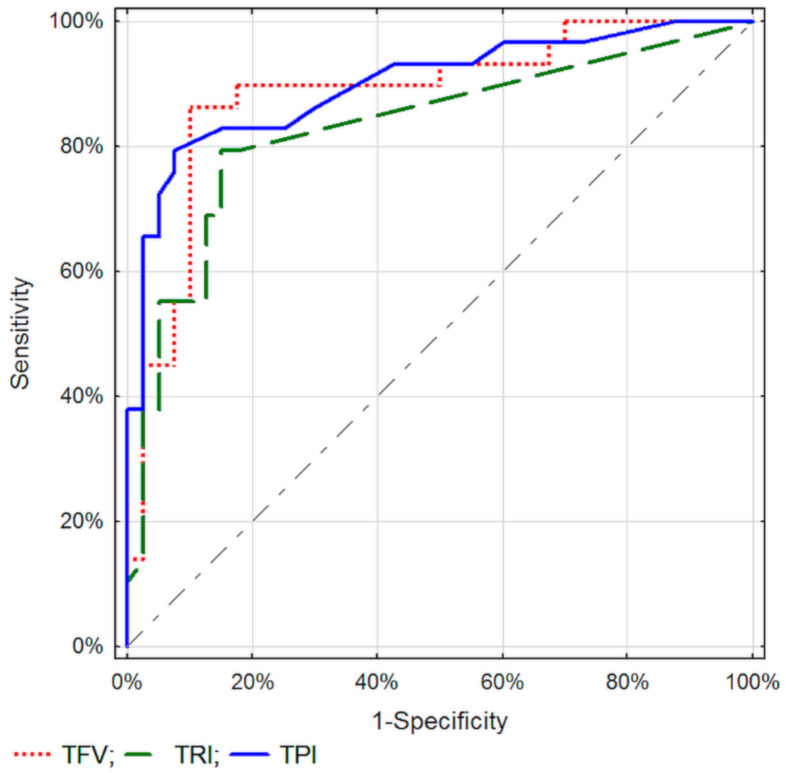
ROC curves for selected tissue perfusion parameters in differentiating between malignant and inflammatory pancreatic lesions.

**Figure 2:**
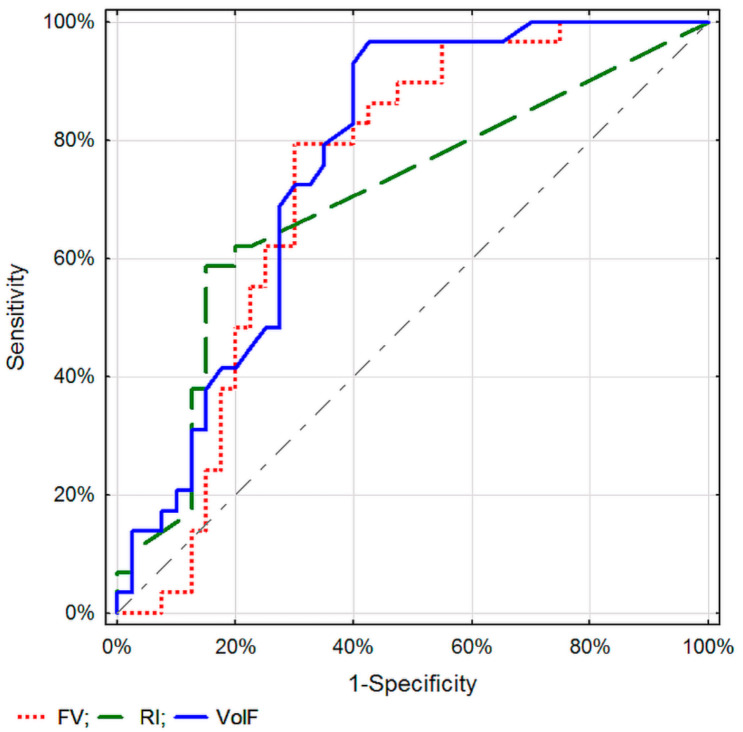
ROC curves for selected parameters of blood flow assessment in a single tumor vessel in differentiating between malignant and inflammatory pancreatic lesions.

The discriminatory properties of the considered parameters within the given method, DTPM or SVFM, did not differ significantly (*p* > 0.01) (Figure 1 and Figure 2).

**Figure 3:**
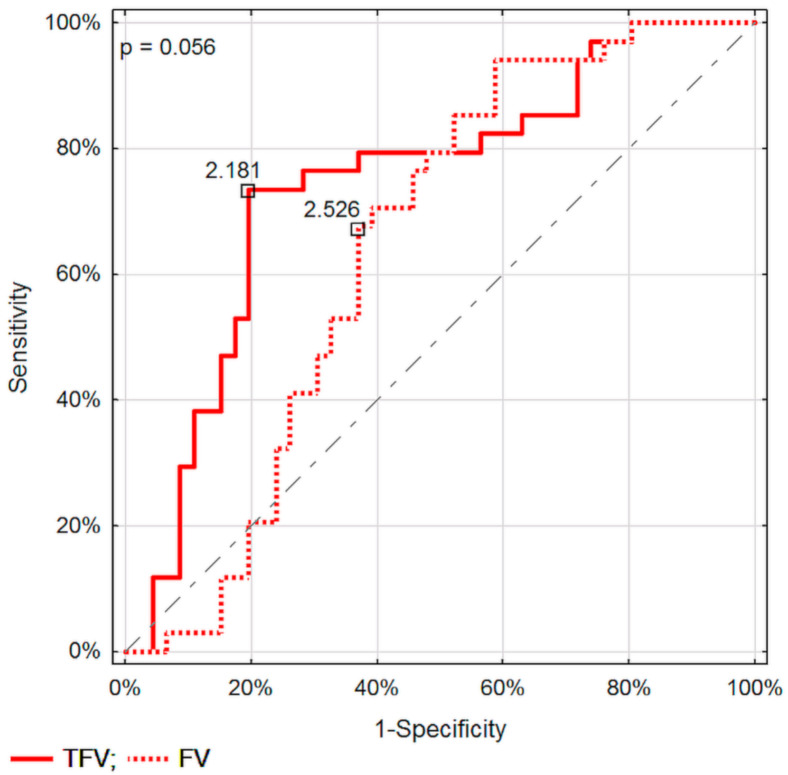
Comparison of ROC curves in differentiating between malignant and inflammatory pancreatic lesions for flow velocity assessed in DTPM and SVFM.

**Figure 4:**
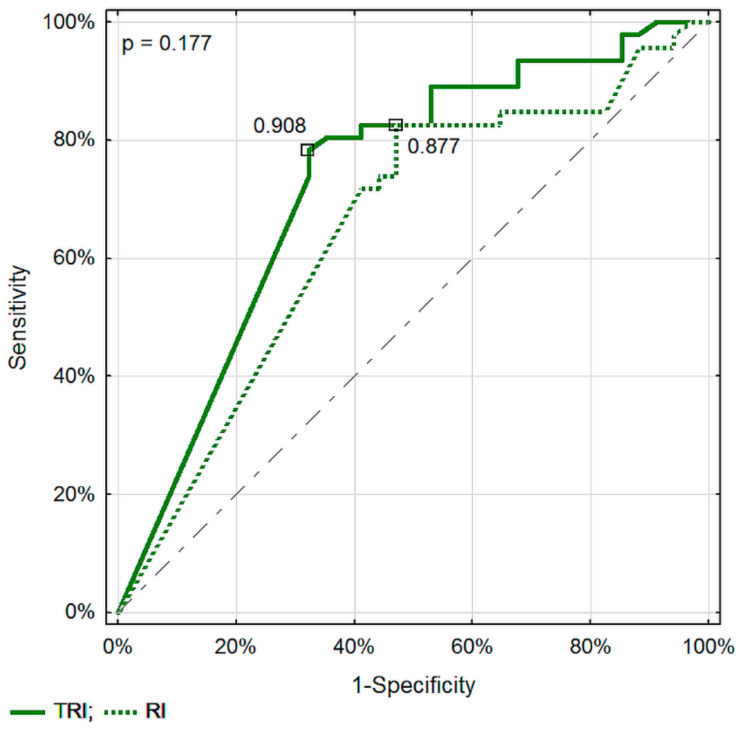
Comparison of ROC curves in differentiating between malignant and inflammatory pancreatic lesions for the resistive index evaluated in the DTPM and SVFM methods.

**Figure 5:**
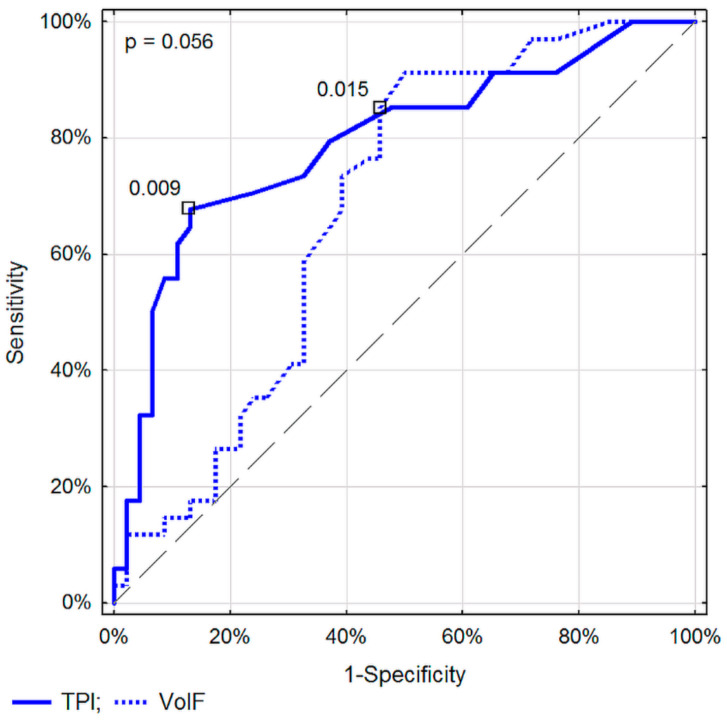
Comparison of ROC curves in differentiating between malignant and inflammatory pancreatic lesions for TPI and svVolF.

Assessment of TPI specific for the DTPM method, and VolF for SVFM, seem to be the best parameters for differentiating pancreatic lesions. No significant differences in recognizing malignant from inflammatory pancreatic lesions between the corresponding parameters of these two Doppler methods were found (Figure 3, Figure 4 and Figure 5).

**Figure 6:**
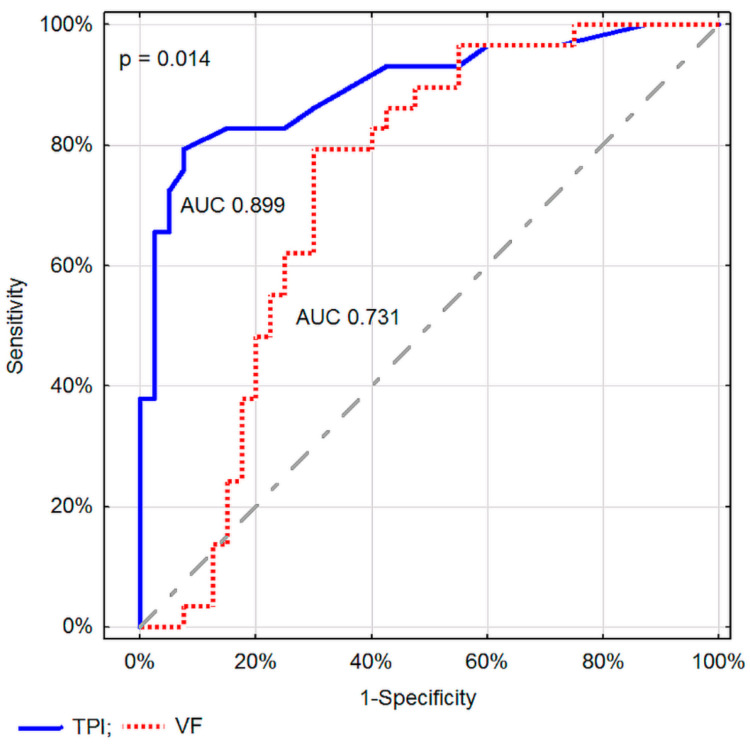
Comparison of ROC curves in differentiating between malignant and inflammatory pancreatic lesions for TPI and FV.

Among the measurements typically assessed as part of the method, the TPI value in DTPM proved to be a significantly better parameter differentiating between inflammatory and malignant pancreatic lesions in comparison to FV in SVFM (*p* = 0.014, Figure 6).

## 4. Discussion

For the first time in the literature, we present the results of comparing the discriminatory properties of ultrasound organ perfusion parameters with single-vessel flow parameters in the differentiation of inflammatory and malignant pancreatic tumors during the EUS examination.

At present, EUS is useful in detecting, characterizing, and acquiring tissue of pancreatic lesions. The current development of contrast-enhanced harmonic EUS and elastography allows for a better characterization of pancreatic lesions. Besides these enhanced EUS imaging techniques, EUS-guided tissue acquisition is currently a routine procedure in establishing the pathological diagnosis of pancreatic tumors [9]. Detection of small pancreatic cancers (PCs), which have a better survival prognosis than large PC, is required to lower mortality rates. EUS is the most sensitive imaging method for diagnosing pancreatic lesions. The high resolution of EUS makes it an especially useful tool to identify small pancreatic lesions that can be missed using other imaging modalities. EUS should thus be performed in patients with obstructive jaundice in whom computed tomography (CT) or magnetic resonance imaging (MRI) does not definitely detect a pancreatic lesion. The usefulness of EUS for screening individuals at high risk of PC, including those with intraductal papillary mucinous neoplasms (IPMNs) and familial PC, appears to be more and more justified. Contrast-enhanced EUS can facilitate differential diagnosis of small solid pancreatic lesions as well as malignant cystic tumors. Additionally, EUS-FNA can provide samples of small pancreatic lesions [10].

Due to the common availability of ultrasound and the standard of diagnosis of pancreatic tumors in the EUS, Doppler assessment of these changes is not difficult. We have previously shown that perfusion parameters of solid pancreatic tumors can be useful in differentiating between malignant and inflammatory lesions. The majority of ultrasound devices currently do not have the possibility of tissue perfusion assessment despite advanced Doppler and non-Doppler methods of blood flow evaluation.

In our previous research, we used external PixelFlux software that allows assessment of tissue perfusion based on movie sequences with a recorded color-coded blood flow using the color Doppler option [7]. Tissue flow velocity evaluated by DTPM proved to be a helpful parameter differentiating between malignant and inflammatory pancreatic tumors with a sensitivity of 83% and specificity of 86% (AUC 0.852; *p* < 0.001).

However, adequate Doppler flow assessment requires a proper correction of the Doppler wave angle to the vessel axis, and a correction above 60° results in a significant falsification of the result. Unlike the assessment of kidney cortex perfusion, where the test is performed in the region of vessels running straight to the transducer, and angle correction is not needed, and such an assumption for pancreatic tumors is frequently not feasible [10,11,12]. Therefore, it may be justified to question the correctness of such an assessment. On the other hand, flow measurement in a single vessel, with appropriate angle correction, is available in every ultrasound apparatus equipped with a pulsed wave Doppler option. Confirmation of the possibility of differentiating between malignant and inflammatory pancreatic lesions during flow assessment in a single vessel within the tumor would, on the one hand, enable easily available diagnostics of these lesions, and on the other hand, would confirm the validity of tissue perfusion assessment as long as the diagnostic properties of DTPM are not worse than SVFM.

The possibilities of differentiating focal pancreatic lesions with the help of tissue perfusion parameters are constantly evolving. The combination of quantitative-elastography endoscopic ultrasound and contrast-enhanced harmonic endoscopic ultrasound (CH-EUS) is helpful in the differential diagnosis of solid pancreatic tumors by providing complementary information. This combination, however, does not significantly raise the diagnostic accuracy of either of the techniques performed alone [13]. Current imaging methods are limited in their ability to differentiate between PC and non-neoplastic pancreatic lesions [14]. Recently, CH-EUS was observed to aid in imaging of parenchymal perfusion and microvessels in malignant pancreatic masses [15,16]. Yamashita et al.’s meta-analysis proved that CH-EUS with qualitative analysis of the enhancement pattern is helpful in diagnosing PC with a high sensitivity of 93% and specificity of 80%, regardless of the type of contrast agent used [17]. Although contrast-based ultrasound imaging methods are considered non-invasive, they require intravenous contrast administration, which can result in adverse effects, prolonged diagnostic time, and have low repeatability [18]. Recently, the new ultrasound machines have been increasingly offering the option of microvascular imaging, which does not require contrast administration, allows for a qualitative assessment of organ perfusion, and seems to be a future alternative to ultrasound contrast examinations [19,20,21].

In the presented study, in order to evaluate the flow in a single tumor vessel, we used the option of the PixelFlux program, which in addition to assessing perfusion in a given region of interest, also allows for the assessment of blood flow velocity and volume in a single vessel, corresponding to the Duplex Doppler (Color Doppler + Pulsed Wave Doppler) mode. The parameters of Doppler perfusion and blood flow in a single vessel that we assessed enabled adequate differentiation of malignant and inflammatory pancreatic lesions. Despite the lack of angle correction, perfusion parameters proved to be better in this diagnosis than the corresponding parameters of blood flow in a single vessel (difference at the level of statistical significance). In everyday diagnostics, the flow velocity (FV) is the parameter most frequently and most easily assessed in a single vessel. In addition, accurate measurement of the small vessel diameter to calculate volume flow is time consuming and extremely difficult during the EUS, which rather prevents the assessment of VolF from being useful in everyday practice. Meanwhile, a comparison of parameters typically evaluated in the tested Doppler methods, i.e., TPI in DTPM and FV in SVFM, showed a significant advantage of organ perfusion examination over classic Doppler flow evaluation. Taking together a shorter and easier Doppler examination in time of EUS and significantly better differentiating properties, we recommend using the DTPM method with the tissue perfusion intensity (TPI) assessment below 0.009 cm/s for recognizing malignant pancreatic cancer tumors.

To objectify the examinations and to avoid flow velocity measurement errors associated with a lack of (in DTPM) or excessive angle correction (>60°, in SVFM), we evaluated the resistive index (RI) both in the perfusion method (TRI) and in the single tumor vessel (RI). The RI value is not dependent on the Doppler angle correction. Eventually, this parameter also proved to be useful in differentiating pancreatic lesions, and evaluation by the perfusion method provided slightly better discriminatory properties. This confirms the usefulness of DTPM in the differential diagnosis of solid pancreatic lesions and also reduces the importance of the lack of angle correction in this method.

Despite the promising results, the work presented has several limitations. Although the perfusion parameters did not differ significantly from those corresponding measured in a single vessel, this study excluded lesions without a possibility to identify a single vessel with a known flow direction, or with a relatively large vessel running through the lesion. In these cases, perfusion assessment could still be performed. Another limitation for evaluating the flow measurement in a single vessel is the secondary analysis of the already recorded movie sequences but not real-time testing, which often reduces the diagnostic value of this method. Eventually, both the size of the study group and secondary assessment of blood flow in a single vessel does not allow recommendation of this method in differentiating inflammatory and malignant pancreatic lesions in the general population. On the other hand, the results presented by us can be the basis for their verification in a prospective study in a larger group of patients.

## 5. Conclusions

Ultrasound Doppler parameters of blood flow assessment are useful in the differential diagnostics of malignant and inflammatory pancreatic lesions. The dynamic ultrasound tissue perfusion measurement seems to have better discriminatory properties in the differentiation of solid pancreatic lesions than the Doppler blood flow examination in the single vessel within the tumor.

## Figures and Tables

**Table 1:** Comparison of Doppler examination results of malignant and inflammatory pancreatic lesions.

	Neoplastic Lesions (*n* = 40)		Inflammatory Lesions (*n* = 29)		Significance (*p*)
TFV [cm/s]	1.422	±0.742	2.653	±0.733	<0.001
TRI	1.000	(1.000–1.000)	0.776	(0.601–0.880)	<0.001
TPI [cm/s]	0.004	(0.001–0.007)	0.016	(0.010–0.024)	<0.001
FV [cm/s]	1.592	(0.953–3.148)	3.397	(2.696–4.350)	0.001
RI	1.000	(1.000–1.000)	0.843	(0.648–1.000)	0.002
VolF [mL/s]	0.010	(0.004–0.035)	0.032	(0.022–0.059)	<0.001

FV—single vessel flow velocity; RI—single vessel resistive index; VolF—single vessel volume flow; TFV—tissue flow velocity; TPI—tissue perfusion intensity; TRI—tissue resistive index.

**Table 2:** Discriminatory properties of Doppler parameters in the differentiation of malignant from inflammatory pancreatic lesions.

	Nadir Value	Sensitivity	Specificity	ACC	AUC	Significance (*p*)
TFV [cm/s]	2.181	0.862	0.900	0.884	0.883	<0.001
TRI	0.909	0.850	0.793	0.862	0.831	<0.001
TPI [cm/s]	0.009	0.793	0.925	0.870	0.899	<0.001
FV [cm/s]	2.526	0.793	0.700	0.739	0.731	<0.001
RI	0.877	0.850	0.586	0.739	0.698	0.003
VolF [mL/s]	0.015	0.931	0.600	0.739	0.765	<0.001

ACC—accuracy; AUC—area under curve; FV—single vessel flow velocity; RI—single vessel resistive index; VolF—single vessel volume flow; TFV—tissue flow velocity; TPI—tissue perfusion intensity; TRI—tissue resistive index.

## Data Availability

The data generated and analyzed during this study are available from the corresponding author upon reasonable request.

## References

[1] Mario C., Marilisa F., Kryssia I.R.C., Pellegrino C., Ginevra C., Chiara M., Alberto B., Antonio N., Gioacchino L., Tiziana M. (2018). Epidemiology and risk factors of pan-creatic cancer. Acta Biomed..

[2] Drouillard A., Manfredi S., Lepage C., Bouvier A.-M. (2018). Épidémiologie du cancer du pancréas. Bull. Cancer.

[3] McGuigan A., Kelly P., Turkington R.C., Jones C., Coleman H.G., McCain R.S. (2018). Pancreatic cancer: A review of clinical diagnosis, epidemiology, treatment and outcomes. World J. Gastroenterol..

[4] Ilic M., Ilic I. (2016). Epidemiology of pancreatic cancer. World J. Gastroenterol..

[5] Stark A.P., Sacks G.D., Rochefort M.M., Donahue T.R., Reber H.A., Tomlinson J.S., Dawson D.W., Eibl G., Hines O.J. (2016). Long-term survival in patients with pan-creatic ductal adenocarcinoma. Surgery.

[6] Dyrla P., Lubas A., Gil J., Niemczyk S. (2016). Doppler tissue perfusion parameters in recognizing pancreatic malignant tumors. J. Gastroenterol. Hepatol..

[7] Dyrla P., Gil J., Kosik K., Schneditz D., Saracyn M., Niemczyk S., Lubas A. (2019). Doppler tissue perfusion measurement is a sensitive and specific tool for a differentiation between malignant and inflammatory pancreatic tumors. PLoS ONE.

[8] Scholbach T., Scholbach J., Di Martino E. (2009). Dynamic Sonographic Tissue Perfusion Measurement. J. Med. Ultrasound.

[9] Nakai Y., Takahara N., Mizuno S., Kogure H., Koike K. (2019). Current Status of Endoscopic Ultrasound Techniques for Pancreatic Neoplasms. Clin. Endosc..

[10] Yoshida T., Yamashita Y., Kitano M. (2019). Endoscopic Ultrasound for Early Diagnosis of Pancreatic Cancer. Diagnostics.

[11] Lubas A., Kade G., Saracyn M., Niemczyk S., Dyrla P. (2018). Dynamic tissue perfusion assessment reflects associations between anti-hypertensive treatment and renal cortical perfusion in patients with chronic kidney disease and hypertension. Int. Urol. Nephrol..

[12] Scholbach T. (2012). Dynamic Tissue Perfusion Measurement—Basics and Applications. Sonography.

[13] Iglesias-Garcia J., Lindkvist B., Lariño-Noia J., Abdulkader-Nallib I., Dominguez-Muñoz J.E. (2017). Differential diagnosis of solid pan-creatic masses: Contrast-enhanced harmonic (CEH-EUS), quantitative-elastography (QE-EUS), or both?. United Eur. Gastroenterol. J..

[14] Mei S., Wang M., Sun L. (2019). Contrast-Enhanced EUS for Differential Diagnosis of Pancreatic Masses: A Meta-Analysis. Gastroenterol. Res. Pract..

[15] Kitano M., Kudo M., Sakamoto H., Nakatani T., Maekawa K., Mizuguchi N., Ito Y., Miki M., Matsui U., von Schrenck T. (2008). Preliminary study of contrast-enhanced har-monic endosonography with second-generation contrast agents. J. Med. Ultrason..

[16] Li Y., Jin H., Liao D., Qian B., Zhang Y., Xu M., Han S. (2019). Contrast-enhanced harmonic endoscopic ultrasonography for the differential diagnosis of pancreatic masses: A systematic review and meta-analysis. Mol. Clin. Oncol..

[17] Yamashita Y., Shimokawa T., Napoléon B., Fusaroli P., Gincul R., Kudo M., Kitano M. (2019). Value of contrast-enhanced harmonic endo-scopic ultrasonography with enhancement pattern for diagnosis of pancreatic cancer: A meta-analysis. Dig. Endosc..

[18] Emanuel A.L., Meijer R.I., Van Poelgeest E., Spoor P., Serné E.H., Eringa E. (2019). Contrast-enhanced ultrasound for quantification of tissue perfusion in humans. Microcirculation.

[19] Zhang D., Xin X.J., Mu J., Mao Y.R., Zhang S. (2019). Comparative analysis of superb microvascular imaging and contrast-enhanced ultrasound in diagnosis of small renal masses. Zhonghua Yi Xue Za Zhi.

[20] Ohno Y., Fujimoto T., Shibata Y., Ohno Y. (2016). A New Era in Diagnostic Ultrasound, Superb Microvascular Imaging: Preliminary Results in Pediatric Hepato-Gastrointestinal Disorders. Eur. J. Pediatr. Surg..

[21] He M.-N., Lv K., Jiang Y.-X., Jiang T.-A. (2017). Application of superb microvascular imaging in focal liver lesions. World J. Gastroenterol..

